# Deciphering the molecular networks of 3-methylcholanthrene-induced clear cell renal cell carcinoma through multi-omics integration

**DOI:** 10.1038/s41598-025-34526-x

**Published:** 2026-01-07

**Authors:** Yuzhe Su, Peihuang Chen, Yaoan Wen, Jiangbin Yang, Yeda Chen, Shaoxing Zhu, Shuyuan Zhan, Song Zheng

**Affiliations:** 1 Department of Urology, Fujian Medical University Union Hospital, Fuzhou, Fujian, 350001 China; 2 Department of Urology, Anxi County Hospital, Quanzhou, Fujian, 362400 China; 3 Dongguan People’s Hospital Biobank, The Tenth Affiliated Hospital of Southern Medical University, Dongguan, Guangdong, 523059 China

**Keywords:** CcRCC, 3-Methylcholanthrene, Machine learning, Molecular docking, Network toxicology, Cancer, Computational biology and bioinformatics, Oncology

## Abstract

Exposure to 3-Methylcholanthrene (3-MC) may be associated with the development and progression of clear cell renal cell carcinoma (ccRCC); however, its underlying molecular mechanisms remain unclear. We conducted differential expression analyses across multiple datasets to identify target genes linked to ccRCC. By integrating SHapley Additive exPlanations (SHAP) with machine learning algorithms, network toxicology and molecular docking, we investigated the binding interactions between 3-MC and these target proteins. A total of 99 genes were identified as potential targets involved in 3-MC-induced ccRCC pathogenesis. Four core genes (GPC3, PIK3C2G, PPARA, and TRPA1) were selected through machine learning approaches. SHAP analysis demonstrated the combined contribution of these genes to the model’s predictive performance. GPC3, PIK3C2G, and PPARA were significantly downregulated, whereas TRPA1 was upregulated (*P* < 0.05). Survival analysis using data from The Cancer Genome Atlas (TCGA) revealed significant differences in patient survival based on the expression levels of these genes. Molecular dynamics simulations validated the structural stability of the interaction between 3-MC and PPARA. These findings suggest that 3-MC promotes ccRCC pathogenesis by targeting specific genes.

**Supplementary Information:**

The online version contains supplementary material available at 10.1038/s41598-025-34526-x.

## Introduction

Renal cell carcinoma (RCC) accounts for approximately 2–3% of all cancers. In 2022, there were approximately 435,000 new cases and 156,000 deaths. It is the most common malignant tumor of the kidney (90%), with a peak incidence in individuals aged 60–70 years (male-to-female ratio of 1.5:1)^1^. RCC can be classified into three major subtypes: papillary, chromophobe, and ccRCC^2^. ccRCC is the most common type of kidney cancer, characterized by high heterogeneity and complexity^3^. The pathogenesis of ccRCC is associated with various risk factors, including established ones such as smoking, obesity, hypertension, and diabetes^4^. Additionally, studies have indicated that environmental pollutants, such as perfluoroalkyl substances^5^ and polycyclic aromatic hydrocarbons (PAHs)^6^ are linked to an increased risk of ccRCC. 3-MC is an environmental compound belonging to the class of PAHs^7^. It is released into the environment through the incomplete combustion of large organic molecules found in wood, tobacco, coal, gasoline, waste incineration, straw burning, coal stove combustion, and chemical products^8^. 3-MC enters the human body via inhalation or diet. It exhibits high lipophilicity and resistance to degradation, leading to accumulation in aquatic sediments and bioaccumulation in animals at higher trophic levels through the food chain, resulting in immunotoxicity, reproductive toxicity, and developmental toxicity^8,9^. Beyond this bioaccumulation effect, two types of carcinogens-heterocyclic amines and polycyclic aromatic hydrocarbons (PAHs)- ingested through high-temperature cooked meat may increase the risk of renal cell carcinoma (RCC), including clear cell renal cell carcinoma (ccRCC)^10^.In breast cancer, 3-MC binds to the aryl hydrocarbon receptor (AHR), regulating the expression of cytochrome P450 (CYP) enzymes. This receptor is involved in the carcinogenic activation of environmental pollutants as well as the biosynthesis and metabolism of estrogens^11^. In renal cells, 3-MC enhances the expression of CYP1A1 and CYP1A2 in rat kidney tissue by regulating the expression of P450 enzymes^12^. Another study demonstrated that simvastatin reduces the carcinogenic effects of 3-methylcholanthrene in renal epithelial cells by inhibiting histone deacetylase 1 and reactivating RhoA^13^. Although there is evidence that 3-MC exposure affects the incidence of clear cell renal cell carcinoma (ccRCC), research on the relationship between 3-MC and ccRCC and its potential mechanisms remains relatively limited, research on the relationship between 3-MC and ccRCC and its underlying mechanisms remains relatively scarce.

The emerging discipline of network toxicology integrates principles from network pharmacology and network biology, facilitating the construction of a comprehensive “toxin-characterization-compound-gene-protein” model to elucidate the mechanistic properties of 3-MC^14^. Integrating network toxicology and molecular docking facilitates the translation of complex mechanisms involving multicomponent and multitarget toxins into intuitive graphical models. This enables systematic analysis of target protein interactions and prediction of the molecular mechanisms by which toxins contribute to disease^15^. Our findings elucidate the mechanisms by which 3-MC exposure influences the pathogenesis of clear cell renal cell carcinoma (ccRCC) and identify novel therapeutic targets for its treatment. Furthermore, this study deepens our understanding of the interaction between environmental pollutants and cancer, providing significant insights for environmental toxicology research.

## Methods

### Identification of disease-associated targets

This study obtained five ccRCC transcriptomic datasets from the NCBI GEO database, including GSE66270, GSE167573, GSE213324, GSE53757, and GSE251905. The datasets GSE66270, GSE167573, and GSE213324 comprised the training cohort, while GSE53757 and GSE251905 formed the validation cohort. To minimize batch effects and ensure comparability across cohorts, the “SVA” R package was used to model and correct potential confounding factors. This method employs an empirical Bayesian framework to effectively integrate and adjust residual batch variations. Subsequently, principal component analysis (PCA) was performed to visually assess the correction results, confirming the reduction of batch-related variability and enhancing the reliability of downstream analyses.

### Chemical characterization and target identification of 3-methylcholanthrene

The physicochemical properties, biological parameters, and 2D structural information (SMILES: CC1 = C2CCC3 = C2C(= CC4 = C3C = CC5 = CC = CC = C54)C = C1) of 3-MC were retrieved from the PubChem database (https://pubchem.ncbi.nlm.nih.gov/). Based on these data, a multi-platform integration strategy was employed to predict targets.

### Download and analysis of ccRCC-related data from the TCGA database

RNA-seq data (STAR-aligned) from the TCGA-KIRC (Kidney Renal Clear Cell Carcinoma) project were downloaded and organized from the TCGA database (https://portal.gdc.cancer.gov). FPKM-formatted data were extracted and normalized using a log₂(value + 1) transformation. Survival analysis was conducted using the R packages ggplot2(v3.4.4), stats(v4.2.1), significance car (v3.1-0). of *P* < 0.05 was applied P < identify survival differences. differences in survival.

### Differential gene expression analysis

Differential expression analysis was conducted using the limma package. with an adjusted P-value < 0.05 and |log₂FC| ≥ |log₂ fold change| were considered significantly differentially expressed. A volcano plot was generated using ggplot2to visualize the ggplot2 to of differentially expressed genes. Additionally, a heatmap was created to display expression patterns and clustering trends across all samples.

### Weighted gene co-expression network analysis (WGCNA)

This study utilized the “WGCNA” package in R to construct a scale-free weighted gene co-expression network. The analytical pipeline proceeded as follows: (1) Data Preprocessing: top 5,000 genes with exhibiting highest expression variability were selected to minimize noise interference low-abundance transcripts. Hierarchical clustering based on sample expression profiles was performed to identify and remove outlier samples, ensuring the robust inference. (2) Adjacency Matrix Construction: adjacency matrix was generated by calculating the Pearson coefficients for all gene pairs. (3) Soft Threshold Optimization: fit index was evaluated across a predefined gradient of β range The minimum β value that enabled the network allowed approximate a scale-free distribution with R² ≥ 0.8 was selected as the optimal soft threshold. (4) Topological Overlap and Module Identification: transformed into a Topological Overlap Matrix (TOM). Gene hierarchical clustering was performed based on TOM dissimilarity. Initial modules were identified using the dynamic tree cut algorithm (minModuleSize = 50, deepSplit = 2). Modules with eigengene correlations > 0.75 were merged > = 0.25), resulting in robust co-expression modules. (5) Module-Trait Association: between module eigengenes and the tumor-normal status. Modules with > 0.5 and *P* < 0.05 were considered significantly associated with the ccRCC phenotype. (6) Hub Gene Identification: screened based on the highest connectivity, identified a module membership (kME) > 0.8. were further filtered through differential These genes analysis. Ultimately, genes that were significantly differentially expressed and highly were both with their modules were integrated to form a candidate gene set for ccRCC.

### Identification of CcRCC targets associated with 3-MC

To precisely identify the key effector molecules of 3-MC in ccRCC, this study integrated three gene sets: differentially expressed genes (DEGs), WGCNA hub genes, and predicted 3-MC target genes. A triple intersection analysis was conducted in the R environment, and the overlapping genes were defined as “3-MC-ccRCC target genes.” This overlap was visualized using a Venn diagram generated with the “VennDiagram” package. core target gene set was then imported into the STRING database (v11.5, confidence score ≥ 0.4, species limited to “Homo sapiens”) construct a protein-protein interaction (PPI) network. Finally, Cytoscape 3.10 was employed topological analysis and visualization to elucidate the interactions among target genes and their expression characteristics in tumors.

### Functional enrichment analysis

Functional enrichment analysis was conducted using the “clusterProfiler” package, encompassing Gene Ontology (GO) annotations and Kyoto Encyclopedia of Genes and Genomes (KEGG) pathway annotations. This analysis aimed to elucidate the potential mechanisms by which 3-MC contributes to the initiation and progression of ccRCC.

### Identification of core genes using machine learning techniques

An integrated machine learning prediction framework incorporating multiple algorithms was developed to identify diagnostic biomarkers for ccRCC associated with 3-MC exposure. The specific workflow was as follows: (1) Based on transcriptomic expression profiles from the discovery cohort, eleven classical supervised learning algorithms were applied, including LASSO, Support Tensor Machine (STM), Random Forest (RF), glmBoost, Stepwise Generalized Linear Model (StepGLM),Ridge Regression, Elastic Net (Enet), Gradient Boosting Machine (GBM), Linear Discriminant Analysis (LDA), Extreme Gradient Boosting (XGBoost), and Naive Bayes, generating a total of 127 independent prediction models. (2) Stratified sampling combined with 5-fold cross-validation was employed to optimize hyperparameters, ensuring a balanced class distribution between the training set and the internal validation set. (3) Models were rigorously evaluated using core metrics, including the Area Under the Receiver Operating Characteristic Curve (AUC), Accuracy, and F1 score. (4) A stacked ensemble strategy was used to integrate the best-performing single models, further enhancing predictive robustness. (5) Feature genes were extracted from the final high-confidence ensemble model (AUC > 0.9), ranked based on their frequency of appearance across multiple cross-validation iterations, and selected as candidate core diagnostic biomarkers.

### Machine learning model interpretation

Given the inherent complexity of complex machine learning models, the SHAP​​ algorithm was introduced to quantify the marginal contribution of each feature to individual predictions. This approach enhances the transparency and interpretability of the model’s decisions, enabling researchers to identify which genes (features) were most influential in distinguishing between ccRCC and normal groups, as well as the direction of their impact (positive or negative).

### Computational analysis of molecular Docking interactions

The CB-Dock2 server was utilized for blind docking calculations to systematically evaluate the binding characteristics between 3-MC and the core target proteins^16^.

The operational procedure strictly adhered to the established protocol: (1) Protein structures with a resolution of ≤ 2.5 Å were retrieved from the RCSB PDB database, and the ligand 3-MC was uploaded in SDF format; (2) The platform automatically performed protein side-chain repair, hydrogen atom addition, water molecule removal, ligand 3D structure construction, charge assignment, and format conversion; (3) The CurPocket algorithm identified potential cavities, and the FP2 fingerprint was used to screen templates with ligand similarity ≥ 0.4 in the BioLip database. When templates were available, both AutoDock Vina structure-based docking and FitDock template-based docking were initiated simultaneously, with a grid side length set to 25 Å and 20 conformations sampled. The final results were merged based on Vina scores.

### Molecular dynamics simulations

Molecular dynamics simulations were conducted using GROMACS 2022. Ligand parameters were generated with the GAFF2 force field via sobtop_1.0 (dev3.1), and charges were assigned using the RESP method. The receptor protein was parameterized with the AMBER14SB force field. The system was solvated using the TIP3P water model within a cubic water box with a side length of 1 nm^17^. Na⁺ and Cl⁻ ions were added using gmx genion to neutralize the system electrically and maintain a physiological ionic strength of 0.15 M NaCl. Long-range electrostatic interactions were treated using the Particle Mesh Ewald (PME) method with a cutoff distance of 1 nm, and bond constraints were applied using the LINCS algorithm. Prior to simulation, energy minimization was performed, followed by NPT ensemble simulations at 310 K using a Nosé–Hoover thermostat and at 1 bar using a Parrinello–Rahman barostat. The integration time step was 2 fs, and the total simulation duration was 100 ns. Trajectory analyses included root mean square deviation (RMSD), root mean square fluctuation (RMSF), radius of gyration (Rg), and solvent-accessible surface area (SASA). The binding free energy of the complex was calculated using the MM-PBSA method with the g_mmpbsa tool.

### Processing and annotation of single-cell RNA-sequencing data

We obtained the single-cell transcriptome dataset GSE156632 from the NCBI-GEO database and processed it using the standard Seurat 4.3.0 workflow. After importing the raw matrix using Read10X, low-quality cells were filtered according to the following criteria: nFeature between 200 and 8000, nCount greater than 200, excluding the top 3% of cells by count, percent.mt below 15%, and percent.hb below 3%. The data were normalized using LogNormalize, and the top 2000 highly variable genes were selected. Dimensionality reduction was performed using PCA with 10 principal components (npcs = 10), followed by batch correction using Harmony. Visualization was performed using Uniform Manifold Approximation and Projection (UMAP). Manual cell type annotation was performed based on the gene list established in previous study^18^. To validate these manual annotations, seven major human immune reference datasets were integrated, and automated cell type annotation and mapping were performed using SingleR. This analytical process can identify specific cell populations that express key biomarkers.

### Estimation of immune cell infiltration by SsGSEA

Gene expression data were preprocessed by averaging duplicated gene symbols and removing genes with zero expression. Utilizing the previously established four-gene model, TCGA samples were categorized into predicted tumor and predicted normal groups, hereafter referred to as tumor and normal groups for clarity and consistency.

Immune cell infiltration was estimated using the CIBERSORT algorithm based on predefined immune cell signature matrices, whereas single-sample gene set enrichment analysis (ssGSEA), implemented in the GSVA package (R), was applied to evaluate the relative enrichment patterns of immune- and stromal-related functional gene sets.

### Gene set enrichment analysis

Functional enrichment analyses were conducted using the clusterProfiler package, including gene set enrichment analysis (GSEA) based on ranked gene lists and gene set variation analysis (GSVA) to assess pathway activity variations across samples, with KEGG and Gene Ontology (GO) gene sets. Pathways with *p* < 0.05 were considered statistically significant.

### Immunohistochemical analysis based on the human protein atlas

Immunohistochemical (IHC) images and corresponding staining information were obtained from the Human Protein Atlas (HPA, https://www.proteinatlas.org/)^19^. Protein expression patterns in normal and tumor tissues were evaluated using HPA-validated antibodies. Staining intensity, proportion of positive cells, and subcellular localization were assessed according to the annotation criteria provided by the HPA database. The downloaded IHC images were used for qualitative comparison of protein expression between different tissue types.

### RNA extraction and qRT-PCR assays

ccRCC tissues and corresponding adjacent non-tumorous tissues were obtained from ten patients who underwent surgical resection at Fujian Medical University Union Hospital. Total RNA was isolated from these specimens utilizing TRIzol reagent (Invitrogen) in accordance with the manufacturer’s instructions. Complementary DNA (cDNA) synthesis was conducted using the PrimeScript RT Reagent Kit (TaKaRa). Quantitative real-time polymerase chain reaction (qRT-PCR) analysis was carried out employing SYBR Premix Ex Taq (TaKaRa), following the recommended protocols. Gene expression levels were normalized against glyceraldehyde-3-phosphate dehydrogenase (GAPDH) as an internal control. All methods were conducted in accordance with the relevant guidelines and regulations (2023KJT072) approved by the Ethics Committee of Fujian Medical University Union Hospital.

## Result

### Identification of the 3-MC structure and its potential targets

**Fig. 1:**
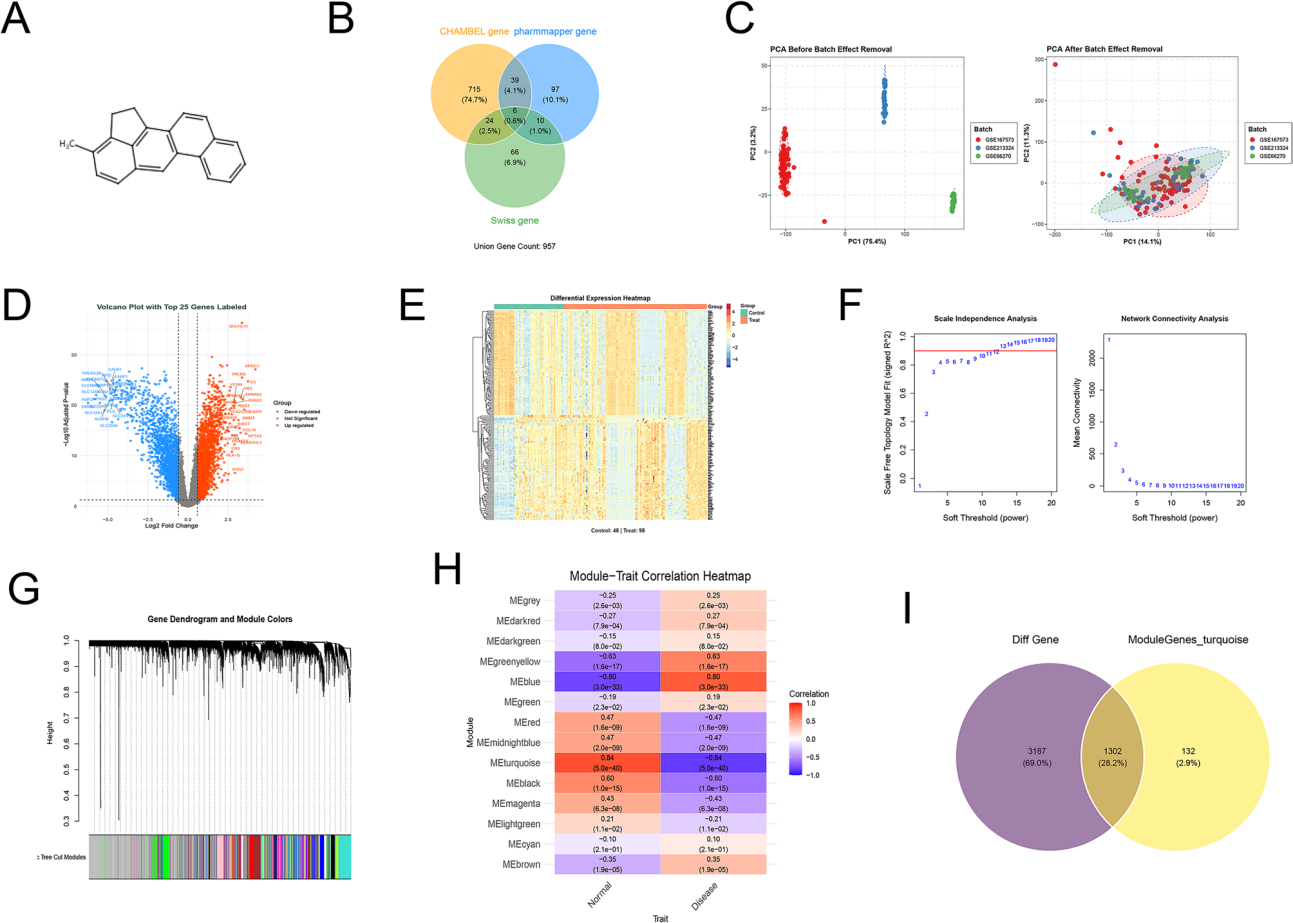
Multilayer Integrated Analysis of 3-MC Targets and ccRCC Targets (**A**) Chemical structure of 3-MC. (**B**) Venn diagram illustrating the target overlap among three databases (ChEMBL, SwissTargetPrediction, and PharmMapper) for 3-MC, identifying 957 consensus target proteins. (**C**) Comparison of the GSE167573, GSE213324, and GSE66270 datasets before and after batch correction. (**D**) Volcano plot of differentially expressed genes (DEGs) between ccRCC and adjacent normal tissue. Red dots indicate significantly upregulated genes in ccRCC; blue dots indicate significantly downregulated genes (*p* < 0.05, |log₂ fold change| ≥ 0.585); gray dots represent genes with no significant difference in expression. (**E**) Heatmap of the top 200 differentially expressed genes (DEGs) in the sample, with blue indicating low expression and red indicating high expression. (**F**) WGCNA scale-free topology fitting analysis determined a soft-thresholding power β = 13 as the minimum threshold required to construct an approximate scale-free network (R² ≈ 0.85). (**G**) Gene clustering dendrogram based on topological overlap measures. (**H**) Heatmap displaying correlation coefficients and corresponding p-values between modules and clinical traits. (**I**) Identification of 1,302 candidate target genes highly associated with ccRCC, derived from the intersection of differentially expressed genes (purple) and WGCNA module core genes (yellow).

The molecular formula of 3-MC is C₂₁H₁₆, and its structure was retrieved from the PubChem database (Fig. 1A). By integrating data from three online databases—ChEMBL, PharmMapper, and Swiss Target Prediction—we identified 957 potential targets for 3-MC for subsequent analysis (Fig. 1B).We retrieved datasets GSE66270, GSE167573, and GSE213324 from the GEO database to identify potential targets for ccRCC. After removing batch effects and performing systematic normalization, principal component analysis (PCA) confirmed that the normalized expression profiles exhibited substantial spatial overlap (Fig. 1C). Figure 1D and E show the volcano plot of differentially expressed genes and the clustering heatmap in ccRCC, respectively.

During soft threshold selection for constructing the weighted gene co-expression network, the signed R² value of the scale-free topology fit index varied with increasing soft threshold power, as shown (Fig. 1F). We selected a power of 13 because it achieved a high scale-free topology fit (exceeding the recommended threshold of 0.8). Concurrently, the average connectivity decreased with increasing soft threshold power, indicating biologically plausible network sparsity (Fig. 1F). Gene clustering based on topological overlap dissimilarity (TOD) produced a dendrogram illustrating hierarchical clustering structures, with the vertical axis (Height) representing TOD values. Multiple co-expression modules were identified using dynamic tree cutting (Fig. 1G). Module-trait association analysis revealed significant correlations between several modules and ccRCC. The largest module, MEturquoise, showed a significant negative correlation with the ccRCC trait (*r* = −0.84, *p* = 5.0 × 10⁻⁴⁰, Fig. 1H). Intersecting differentially expressed genes with WGCNA module genes yielded 1,302 target genes closely associated with ccRCC (Fig. 1I).

### Role of 3-MC-associated disease targets in CcRCC

**Fig. 2:**
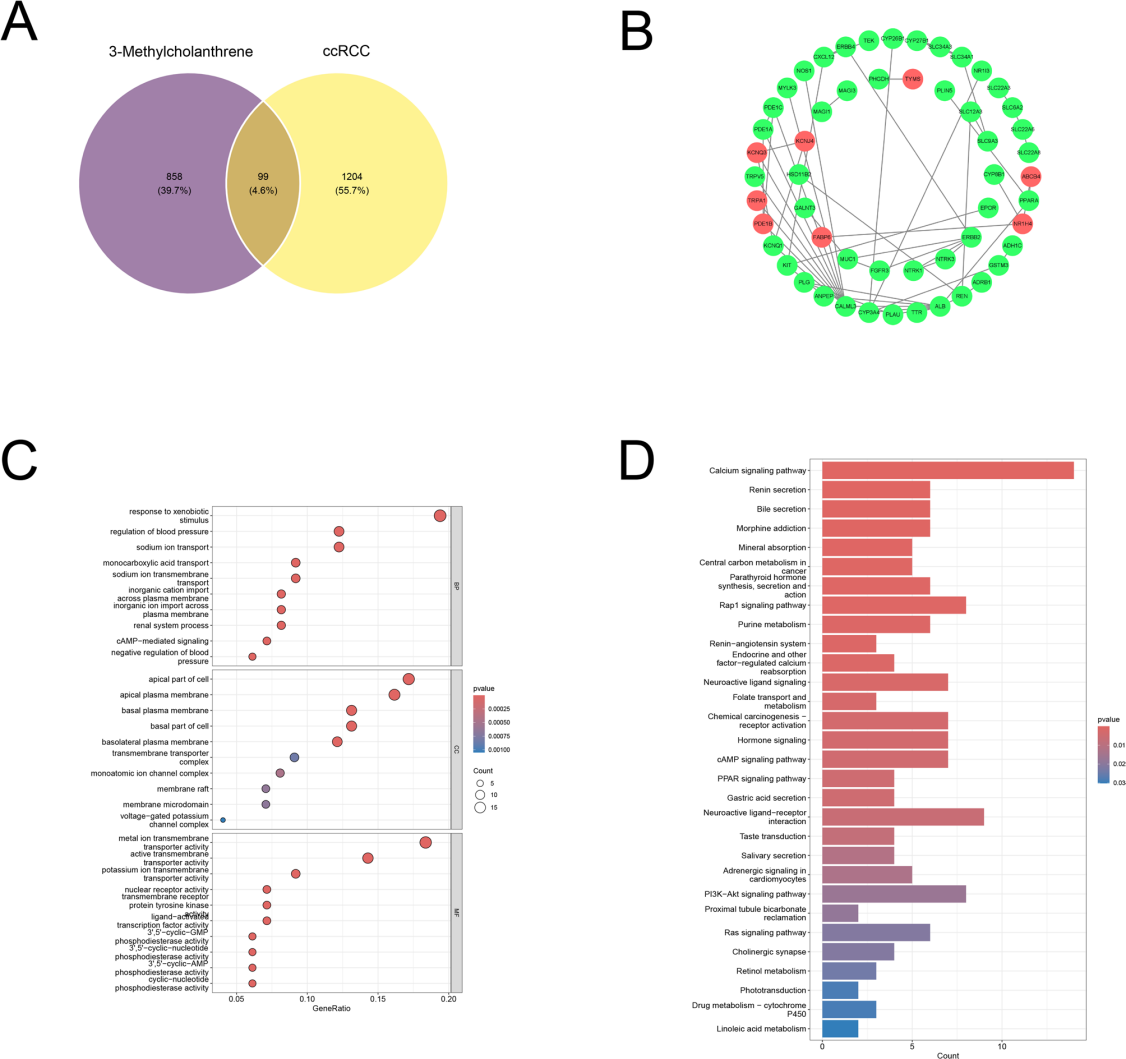
Identification and Enrichment Analysis of Overlapping Target Genes Between 3-MC and ccRCC (**A**) Ninety-nine overlapping genes were identified at the intersection of 3-MC-associated genes (purple) and ccRCC-associated genes (yellow). (**B**) Protein interaction network diagram of overlapping genes: red indicates genes upregulated in ccRCC, blue indicates downregulated genes, and edges represent protein interactions predicted by the STRING database. (**C**) GO functional enrichment analysis of overlapping genes, encompassing biological processes, cellular components, and molecular functions. The x-axis represents the number of enriched genes, while the color gradient indicates corrected p-values, with red denoting higher significance. (**D**) KEGG pathway enrichment analysis of overlapping genes. The x-axis represents the number of enriched genes, while the color gradient reflects the corrected p-values, with red indicating higher significance.

By intersecting the 3-MC-associated gene set with the ccRCC-associated gene set, we identified 99 potential genes involved in the 3-MC-induced molecular network of ccRCC (Fig. 2A). The protein interaction network illustrated the expression levels of these genes in ccRCC cells, with red indicating upregulation and green indicating downregulation (Fig. 2B). We performed GO and KEGG enrichment analysis on 99 candidate genes^20^. Gene Ontology (GO) enrichment analysis revealed significant functional features across three categories: Biological Process (BP), Cellular Component (CC), and Molecular Function (MF). Biological Process: The most significantly enriched terms included responses to external stimuli, regulation of blood pressure, and renal system processes. Key transport-related processes, such as sodium ion transport across membranes and monocarboxylate transport, were also significantly enriched. Cellular Component: Enrichment was observed in membrane structures, including the apical plasma membrane, basolateral plasma membrane, and membrane rafts/microdomains. Specific complexes, such as the voltage-gated potassium channel complex and UniProt ion channel complex, showed significant association. Molecular Function: Major enriched functions included metal ion transmembrane transporter activity, potassium ion transmembrane transporter activity, and nuclear receptor activity. Key enzyme activities, such as 3’,5’-cAMP phosphodiesterase activity and phosphodiesterase activity, were also significantly represented (Fig. 2C). KEGG pathway enrichment analysis revealed substantial involvement of renal and endocrine regulatory pathways in the studied cohort, with calcium signaling exhibiting the highest enrichment level. Additionally, pathways related to mineral absorption, bile secretion, and purine metabolism were implicated (Fig. 2D).

### Identification of core genes in 3-MC-induced CcRCC

**Fig. 3:**
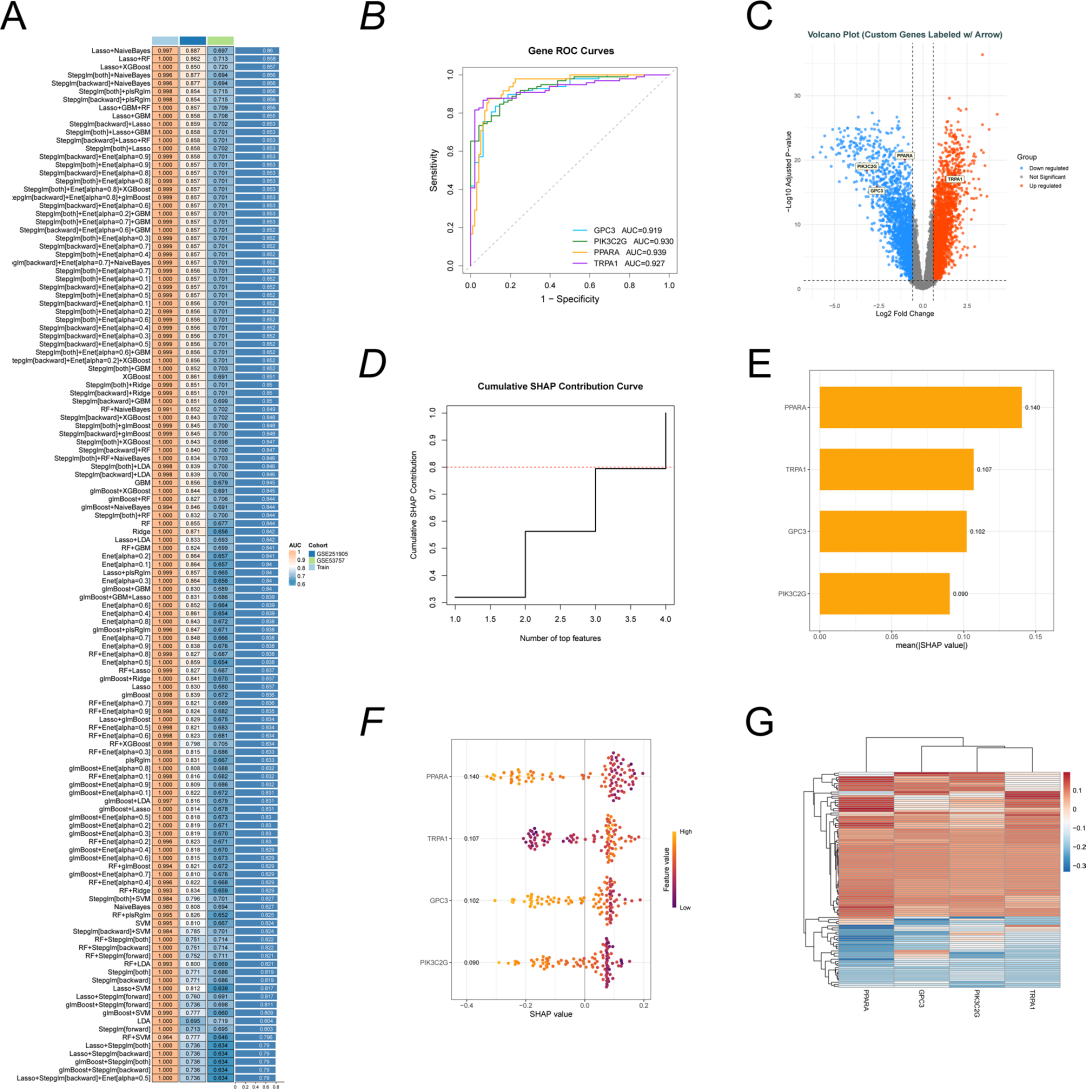
Screening and interpretability analysis of core genes in ccRCC using 127 machine learning models (**A**) Heatmap comparing the performance of multi-cohort models. The left column lists the algorithm names, while the right column shows the corresponding AUC values. Color gradients visually represent differences in prediction performance. The Stepglm[both] + NaiveBayes algorithm was ultimately selected as the target model. (**B**) ROC curves for each core gene in the Stepglm [both] + Naive Bayes prediction model. (**C**) Volcano plot of differentially expressed core genes (p-value < 0.05 and |log₂ fold change| ≥ 0.585). (**D**) Cumulative SHAP contribution curves illustrating the contributions of core genes. (**E**) SHAP contribution bar charts quantifying gene importance. (**F**) SHAP swarm plot: The horizontal axis represents SHAP values, indicating the direction and strength of influence, while the vertical axis lists genes. The color gradient ranges from purple (low expression) to yellow (high expression), illustrating the correlation between gene expression levels and their contributions. (**G**) SHAP value clustering heatmap reveals heterogeneous contribution patterns of core genes across different samples, with colors indicating the magnitude of SHAP values.

We systematically constructed 127 machine learning models to identify core driver genes associated with 3-MC-induced ccRCC (Fig. 3A). Based on both AUC performance and model complexity, the top three models with high AUC values but excessive numbers of integrated genes were excluded. The Stepglm (both) + Naive Bayes model, which ranked fourth in AUC performance, was selected and comprised four core genes (GPC3, PIK3C2G, PPARA, and TRPA1). ROC curve analysis demonstrated that these genes exhibited significant diagnostic value in ccRCC (Fig. 3B). ROC curve and confusion matrix analyses were conducted in two independent validation cohorts, GSE53757 and GSE251905. In the GSE53757 cohort, GPC3 demonstrated the highest diagnostic accuracy (AUC = 0.892), followed by PIK3C2G (AUC = 0.837), TRPA1 (AUC = 0.798), and PPARA (AUC = 0.705). Consistent results were observed in the GSE251905 cohort, where all four genes exhibited moderate diagnostic performance. In this cohort, PPARA (AUC = 0.717) and PIK3C2G (AUC = 0.703) showed slightly higher accuracy than GPC3 (AUC = 0.695) and TRPA1 (AUC = 0.649). The corresponding confusion matrices all demonstrated satisfactory classification accuracy (Supplementary Fig. 1). To visually highlight the differential expression of these core genes, we annotated them on a volcano plot (Fig. 3C). Feature importance analysis based on SHAP values revealed that a small number of features contributed the majority of the model’s predictive power. The cumulative SHAP contribution curve demonstrated a pronounced feature dominance phenomenon in the predictive model, with the top two features accounting for 80% of the total predictive power—reaching the cumulative contribution threshold of 0.8, as marked by the red dashed line (Fig. 3D). SHAP feature importance analysis revealed significant differences in predictive contributions among the four biomarkers. PPARA exhibited the highest mean absolute SHAP value (0.140), indicating it was the most influential predictor in the model. TRPA1 ranked second with a mean SHAP value of 0.107, followed by GPC3 (0.102) and PIK3C2G (0.090) (Fig. 3E). Notably, the direction and magnitude of effects varied by feature: for PPARA and TRPA1, higher gene expression levels consistently correlated with positive SHAP values, meaning they pushed the model’s prediction toward higher-risk or positive outcome categories. Conversely, for GPC3 and PIK3C2G, lower expression levels correlated with positive SHAP values, indicating that their downregulation contributed to increased risk prediction. This clear stratification suggests that these four genes are key drivers in the model’s decision-making process, exerting opposing effects on prediction outcomes (Fig. 3F). Cluster analysis revealed that samples exhibiting similar PPARA and TRPA1 expression patterns tended to cluster together, suggesting potential co-regulatory relationships between them (Fig. 3G).

### 3.4 identify expression and survival differences associated with core genes

**Fig. 4:**
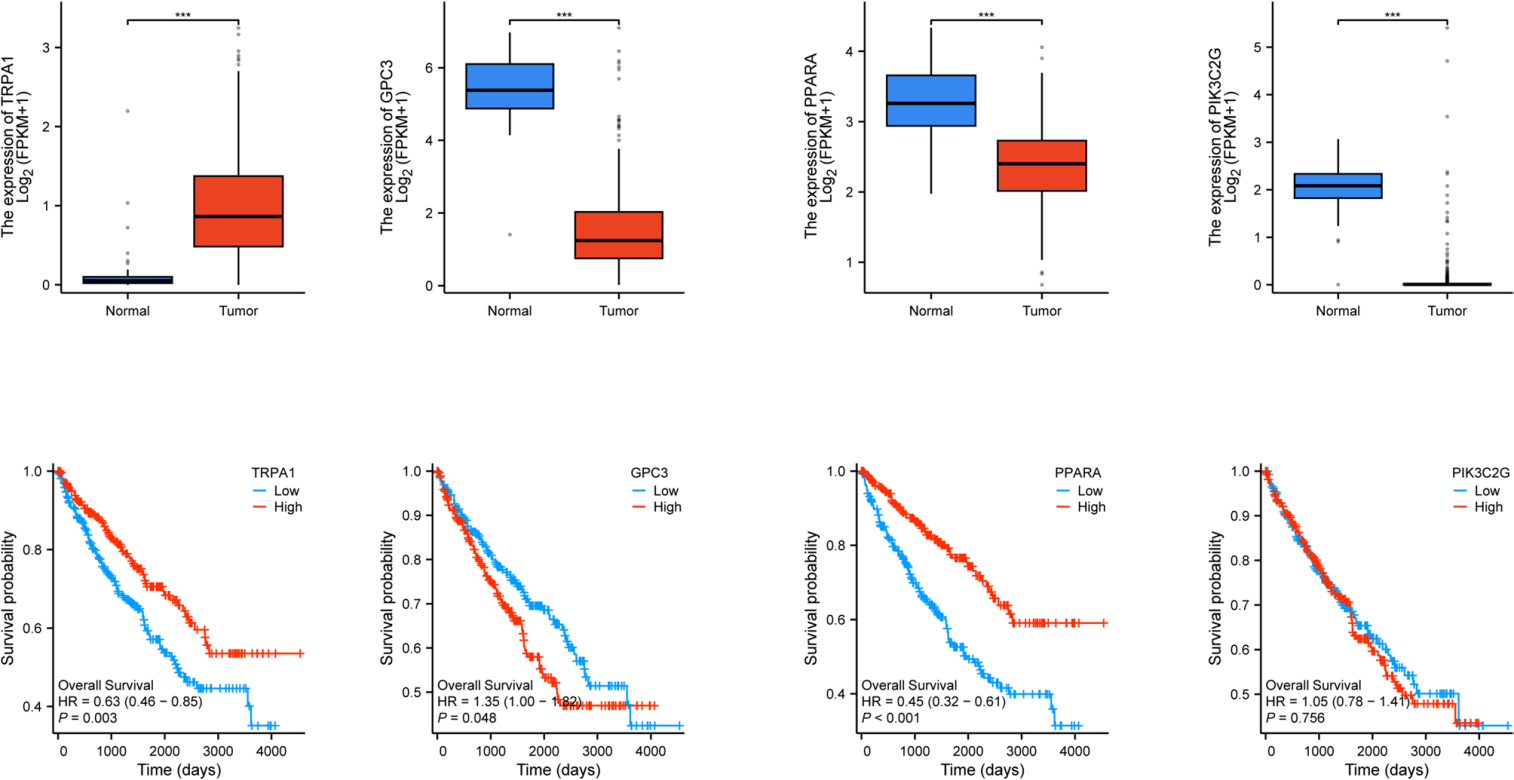
Differential expression and survival analysis based on data from the TCGA database.

Survival analysis based on the TCGA database revealed significant differences in the expression of core genes between disease and normal groups. TRPA1 expression was higher in ccRCC tissues compared to normal tissues, whereas GPC3, PPARA, and PIK3C2G expression levels were lower in ccRCC than in normal tissues (*p **p < 0.01, ***p < 0.001). Furthermore, there were significant differences in survival the high-expression and low-expression rates were observed of high- High expression of of these and PPARA genes was associated with a favorable in ccRCC patients (*P* < 0.05). Conversely, low expression of GPC3 gene was associated with a poor in linked to (*P* < 0.05). PIK3C2G gene expression had no 0.05). Expression of the effect on did not significantly affect conclusion, the identified core genes are closely related to the prognosis of ccRCC (Fig. 4).

### Immune infiltration analysis and SsGSEA results

**Fig. 5:**
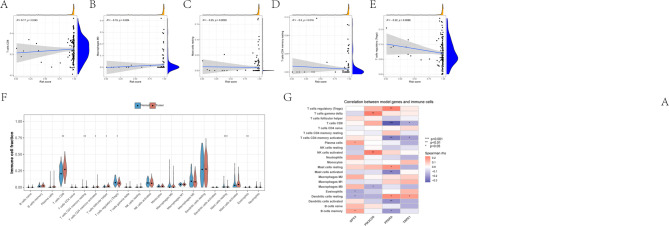
Association between risk score, immune cell infiltration, and model genes (**A–E**) Scatter plots showing the correlations between the risk score and the relative infiltration levels of selected immune cell populations, including CD8⁺ T cells (**A**), M0 macrophages (**B**), resting mast cells (**C**), resting CD4 memory T cells (**D**), and regulatory T cells (Tregs) (**E**). Solid lines represent fitted linear regression trends with 95% confidence intervals. Spearman correlation coefficients (R) and corresponding p values are shown in each panel. Marginal density plots indicate the distribution of risk scores and immune cell fractions. (**F**) Violin plots comparing the relative proportions of immune cell types between the normal and tumor groups. Differences between groups were evaluated statistically, with significance levels indicated above the plots (*p < 0.05, **p < 0.01, ***p < 0.001). (**G**) Heatmap illustrating the Spearman correlation between the expression levels of model genes (GPC3, PIK3C2G, PPARA, and TRPA1) and the infiltration levels of various immune cell types. Color intensity represents the correlation coefficient (rho), with red indicating positive correlations and blue indicating negative correlations. Statistical significance is annotated (*p < 0.05, **p < 0.01, ***p < 0.001).

Immune deconvolution analysis revealed distinct differences in immune cell composition between high- and low-risk groups (Fig. 5F). Several immune cell populations, including CD8⁺ T cells, NK cells, macrophage subsets, dendritic cells, and mast cells, exhibited significantly altered infiltration levels, while other immune cell types showed relatively modest changes. Correlation analysis further revealed that the tumor and normal samples exhibited weak yet statistically significant associations with various immune cell populations, including CD8⁺ T cells, regulatory T cells, resting natural killer (NK) cells, macrophages, and memory CD4⁺ T cells (Fig. 5A–E). This finding indicates a potential link between the model-derived risk and immune cell distribution. In addition, Spearman correlation analysis between the four model genes (GPC3, PIK3C2G, PPARA, and TRPA1) and immune cell infiltration revealed heterogeneous association patterns across various immune cell types (Fig. 5G). Consistently, ssGSEA-based immune functional analysis revealed distinct immune functional profiles between tumor and normal tissues (Supplementary Fig. 2 A). Furthermore, the comprehensive analysis of tumor and normal samples revealed extensive correlations with various immune-related signatures, whereas the associations observed for individual model genes were more heterogeneous (Supplementary Fig. 2B). Taken together, these results indicate that the proposed model is linked to changes in both the composition of immune cells and the functional immune states within the tumor microenvironment.

### Functional pathway analysis based on GSEA and GSVA

To elucidate the biological features underpinning the model-based classification, GSEA and GSVA were conducted utilizing the identical gene expression dataset. GSEA involved ranking genes based on their differential expression between tumor and normal samples, whereas GSVA was employed to assess pathway activity at the level of individual samples, followed by comparative analysis across groups.

**Fig. 6:**
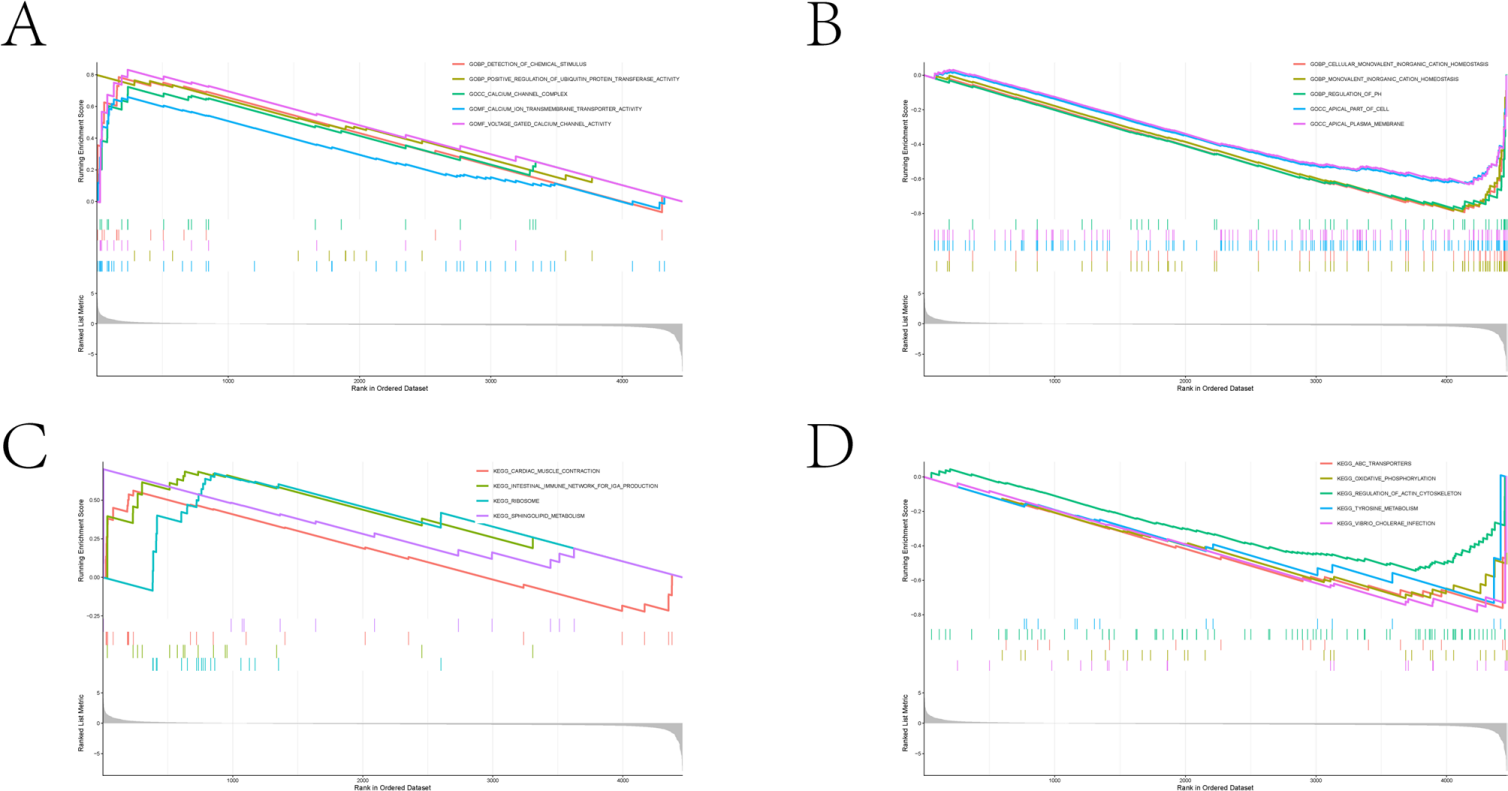
Gene set enrichment analysis (GSEA) of GO terms and KEGG pathways (**A–B**) GSEA showing significantly enriched Gene Ontology (GO) terms, including biological process, molecular function, and cellular component categories. Running enrichment scores are plotted across the ranked gene list, with vertical bars indicating gene set positions. (**C–D**) GSEA of enriched and suppressed KEGG pathways associated with the indicated group. Running enrichment scores are plotted across the ranked gene list, with vertical bars indicating gene set positions.

The GSEA results indicated significant enrichment of pathways associated with ion homeostasis, transmembrane transport, membrane-related functions, and energy metabolism. Specifically, biological processes such as monovalent inorganic cation homeostasis, ion transmembrane transport, and oxidative phosphorylation were predominantly negatively enriched in tumor samples, implying compromised membrane transport capabilities and altered metabolic states (Fig. 6).

In alignment with the GSEA findings, GSVA revealed markedly decreased activity in pathways related to ion transport, cellular ion homeostasis, membrane junction organization, and metabolism within the tumor group. Furthermore, GSVA detected modifications in immune-related and cell cycle–associated pathways, highlighting broad functional disparities between tumor and normal tissues (Supplementary Fig. 3).

Taken together, the consistent outcomes from both GSEA and GSVA analyses suggest that disruptions in membrane transport mechanisms, ionic homeostasis, and metabolic processes constitute key biological characteristics captured by the diagnostic model.

### Single-cell analysis reveals cell type–specific expression patterns and intercellular communication of key genes

**Fig. 7:**
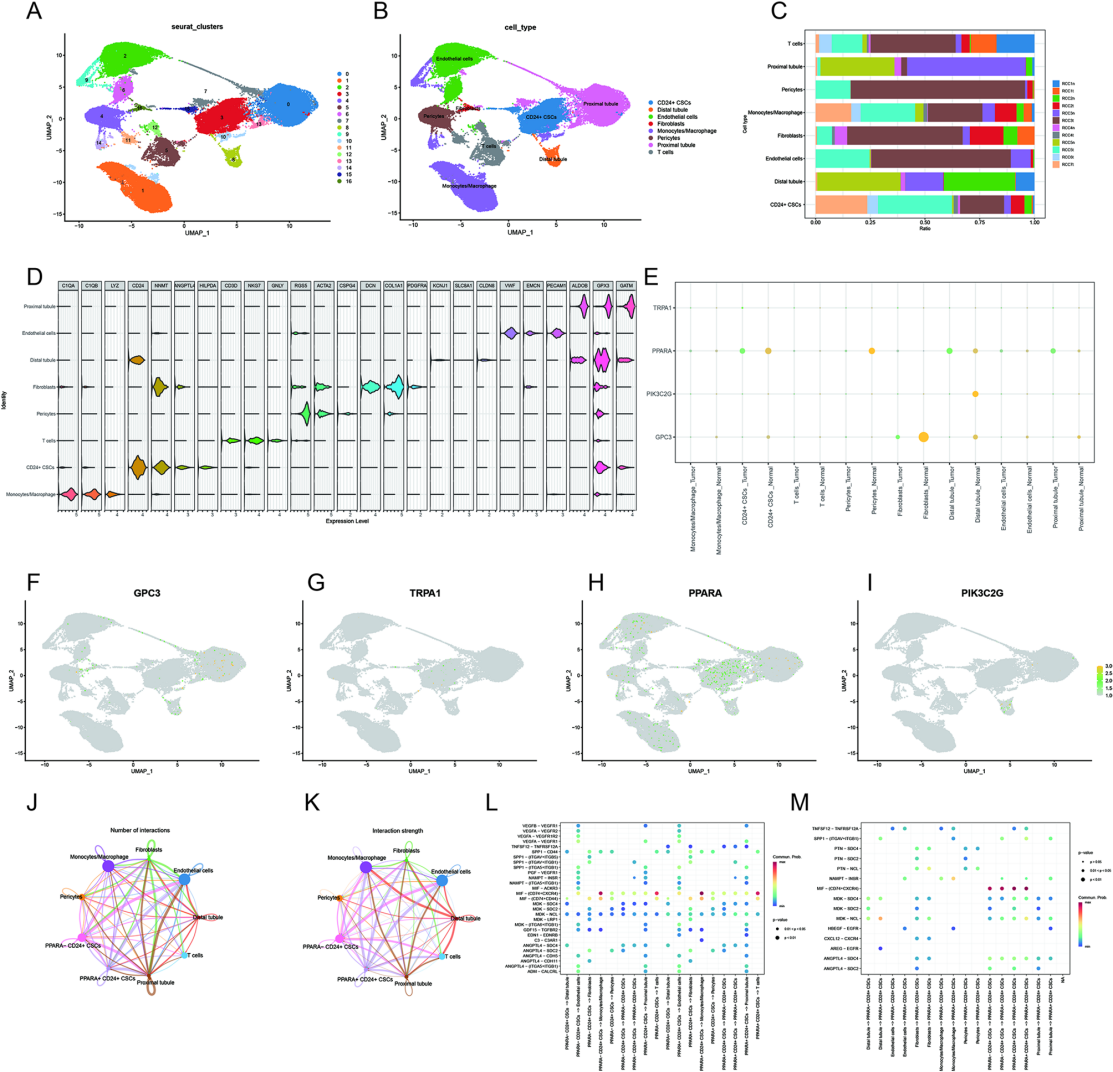
Single-cell analysis of cell type–specific gene expression and intercellular communication (**A**) UMAP visualization of single-cell RNA sequencing data showing the clustering of cells. (**B**) Annotation of cell clusters into eight major cell types, including CD24⁺ cancer stem–like cells (CSCs), distal tubule cells, endothelial cells, fibroblasts, monocytes/macrophages, pericytes, proximal tubule cells, and T cells. (**C**) Relative proportions of the annotated cell types across different GEO samples. (**D**) Dot plot showing the expression patterns of key genes across different cell types, with dot size representing the percentage of expressing cells and color intensity indicating average expression levels. (**E**) Bubble plot illustrating the distribution and relative expression levels of PPARA and GPC3 among the indicated cell populations. (**F–I**) Feature plots displaying the heterogeneous expression of individual key genes projected onto the UMAP embedding. (**J**) Number of predicted cell–cell interactions between PPARA⁺ and PPARA⁻ CD24⁺ CSCs and other cell types. (**K**) Interaction strength of cell–cell communication between PPARA⁺ and PPARA⁻ CD24⁺ CSCs and other cell populations. (**L**) Overview of major ligand–receptor signaling pathways involved in intercellular communication between PPARA⁺ CD24⁺ CSCs and immune or stromal cells. (**M**) Representative ligand–receptor pairs contributing to intercellular communication among CSCs, macrophages, and proximal tubular cells.

To further investigate the cellular distribution and expression characteristics of key genes, we analyzed single-cell RNA sequencing data. UMAP-based cluster analysis revealed distinct cell populations, which were subsequently annotated into eight major cell types: CD24⁺ CSCs, Distal tubules, Endothelial cells, Fibroblasts, Monocytes/Macrophages, Pericytes, Proximal tubules, and T cells (Fig. 7A–B), and the relative proportions of these cell types in different GEO samples were summarized (Fig. 7C).We further analyzed the expression patterns of key genes in different cell types. Quantitative results showed significant cell type-specific expression characteristics (Fig. 7D); bubble plots showed that PPARA and GPC3 had high expression levels in CD24⁺ CSCs and some immune cells (Fig. 7E). Feature maps further illustrated the heterogeneous expression distribution of individual genes in UMAP space, with PPARA showing relative enrichment in CD24⁺ CSCs (Fig. 7F–I). Based on PPARA expression, CD24 + CSCs were divided into two groups. Cell communication analysis revealed the quantity (Fig. 7J) and intensity (Fig. 7K) of interactions between PPARA + and - cells and other cell types. Intercellular communication analysis revealed extensive ligand-receptor interactions among different cell populations. Significant signaling pathways between PPARA⁺CD24⁺ CSCs and immune cells or stromal cells included MIF–CD74/CXCR4, MDK–SDC2/SDC4, and SPP1–ITGAV/ITGB1, as well as angiogenesis-related interactions such as VEGFA/VEGFB–VEGFRs and ANGPTL4–SDC2/SDC4 (Fig. 7L).Further analysis highlighted significant interactions such as TNFSF12–TNFRSF12A, CXCL12–CXCR4, and HBEGF/AREG–EGFR, which primarily occurred between CSCs, macrophages, and proximal renal tubular cells (Fig. 7M).

### Validation of molecular Docking

**Fig. 8:**
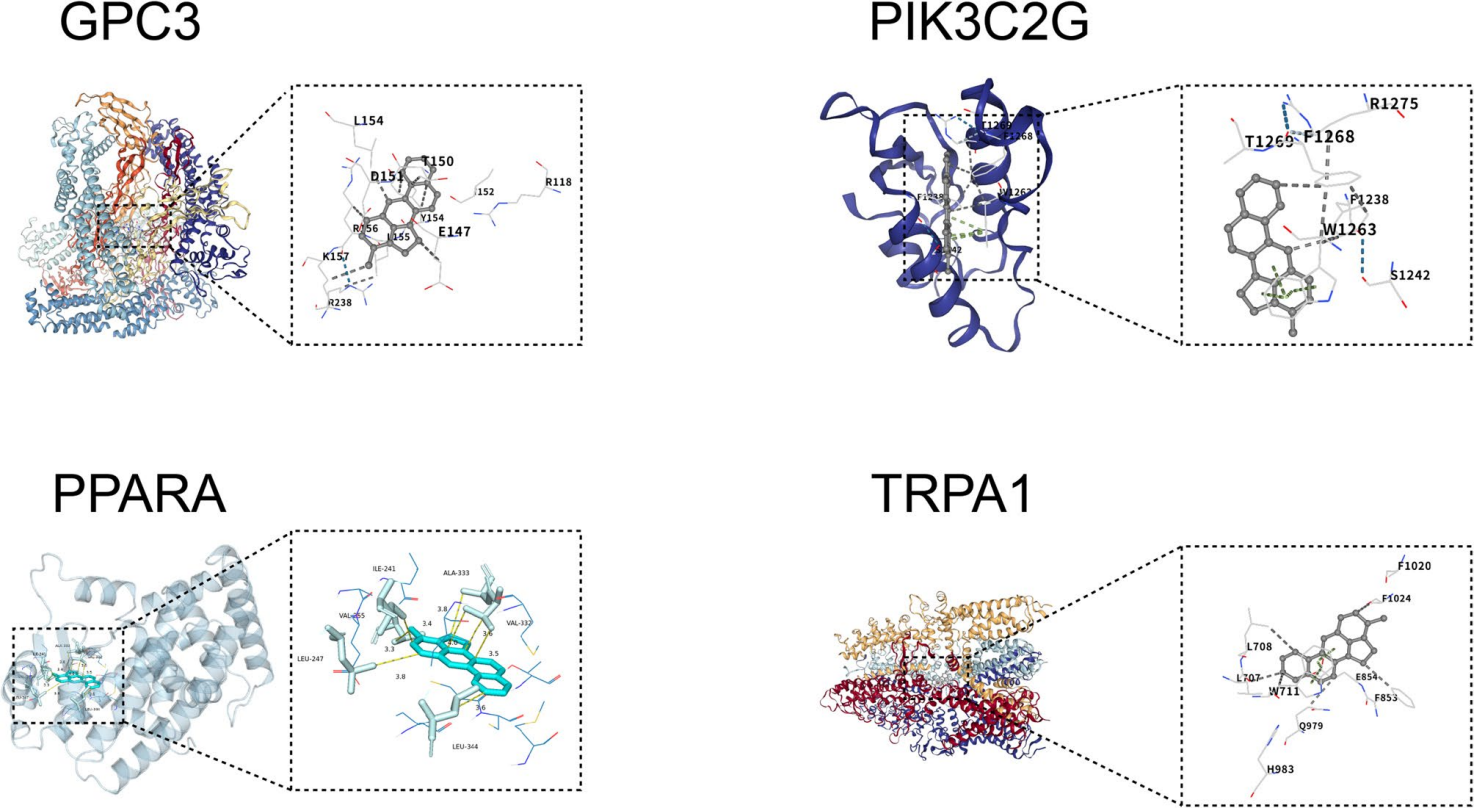
Molecular docking results of 3-MC with target proteins.

To elucidate the interaction patterns between 3-MC and the four core genes, we conducted molecular docking analysis. Within this framework, the binding free energies (ΔG_bind) were consistently below − 5.0 kcal·mol^−1 (Table 1). These values satisfy the established criterion for spontaneous binding (ΔG_bind < 0 kcal·mol^−1) and indicate strong affinity (ΔG_bind < − 5.0 kcal·mol^−1). Visual examination of three-dimensional binding conformations and two-dimensional interaction maps further confirmed the stability of the docking results (Fig. 8). These findings provide valuable insights into the molecular interactions between 3-MC and these key proteins.

**Table 1:** Binding energy between core genes and 3-MC.

Protein	PDB ID or Uniport ID	Ligand	Binding Energy (kcal⋅mol^− 1^)
PPARA	Q15788	3-MC	−10.6
TRPA1	O75762		−9.5
GPC3	P51654		−9.4
PIK3C2G	O75747		−7.7

### Molecular dynamics simulations

**Fig. 9:**
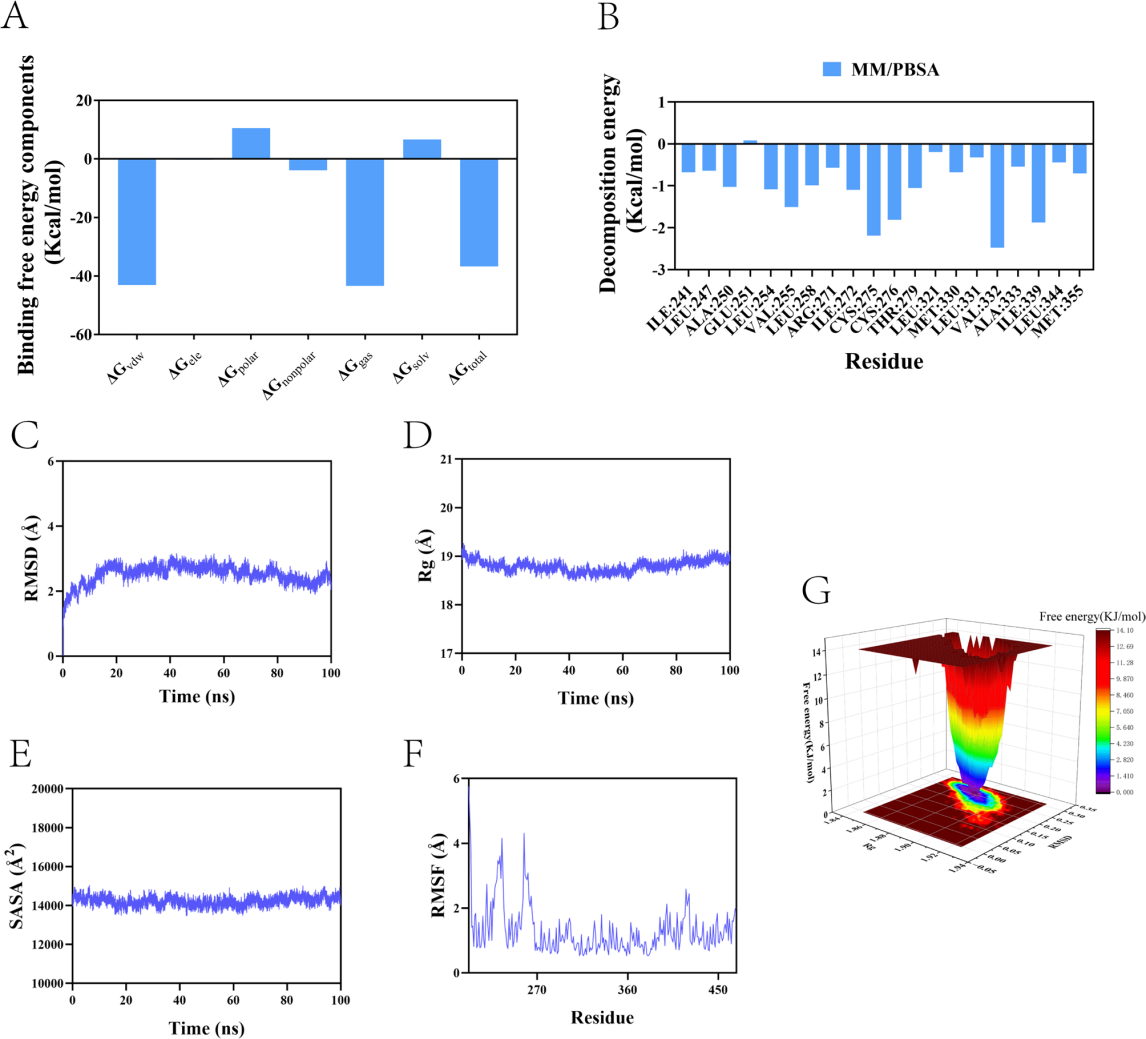
Molecular dynamics simulation of the protein-ligand complex (**A**) Calculation of free combination energy. (**B**) Amino acids that play an important role in the binding of small molecules in the complex system. (**C**) RMSD value of the protein-ligand complex over time. (**D**) Rg value of the protein-ligand complex over time. (**E**) SASA value of the protein-ligand complex over time. (**F**) RMSF value of the protein-ligand complex. (**G**) Free energy landscape.

We further analyzed the molecular docking results using molecular dynamics simulations. Taking the complex formed by PPARA and 3-MC as an example, we calculated the binding free energy of the small molecule and the target protein using the MM/PBSA method based on the binding conformation of the complex (Fig. 9A). The binding free energy of the complex system was − 36.71 Kcal/mol. We further calculated and analyzed the amino acids that significantly contribute to the binding of the small molecule in the complex system. The results showed that residues VAL332, CYS275, ILE339, VAL255, ILE272, THR279, LEU254, and ALA250 had high contribution values ​​in the complex system (Fig. 9B). The root mean square deviation (RMSD) of the complex backbone was used to evaluate the equilibrium of the simulation system, and the results showed that it stabilized at around 2.6 Å after 20 ns (Fig. 9C). The radius of gyration (Rg) remained stable at around 19 Å, indicating that the small molecule-target protein complex did not undergo significant expansion or contraction during motion (Fig. 9D). The structural surface area (SASA) showed no significant change, suggesting that ligand binding had minimal impact on the protein structure (Fig. 9E). The root mean square fluctuation (RMSF) values ​​were relatively low (mostly below 4 Å), indicating lower flexibility and higher stability (Fig. 9F). The Free Energy Landscape (FEL) plot shows the free energy distribution calculated based on RMSD and radius of gyration (Rg) during protein-ligand molecular dynamics simulations. A color gradient is used to represent the free energy levels, gradually decreasing from red (high energy) to blue (low energy) (Fig. 9G).

### PPARA expression is reduced in CcRCC tissues compared with adjacent normal tissues

**Fig. 10:**
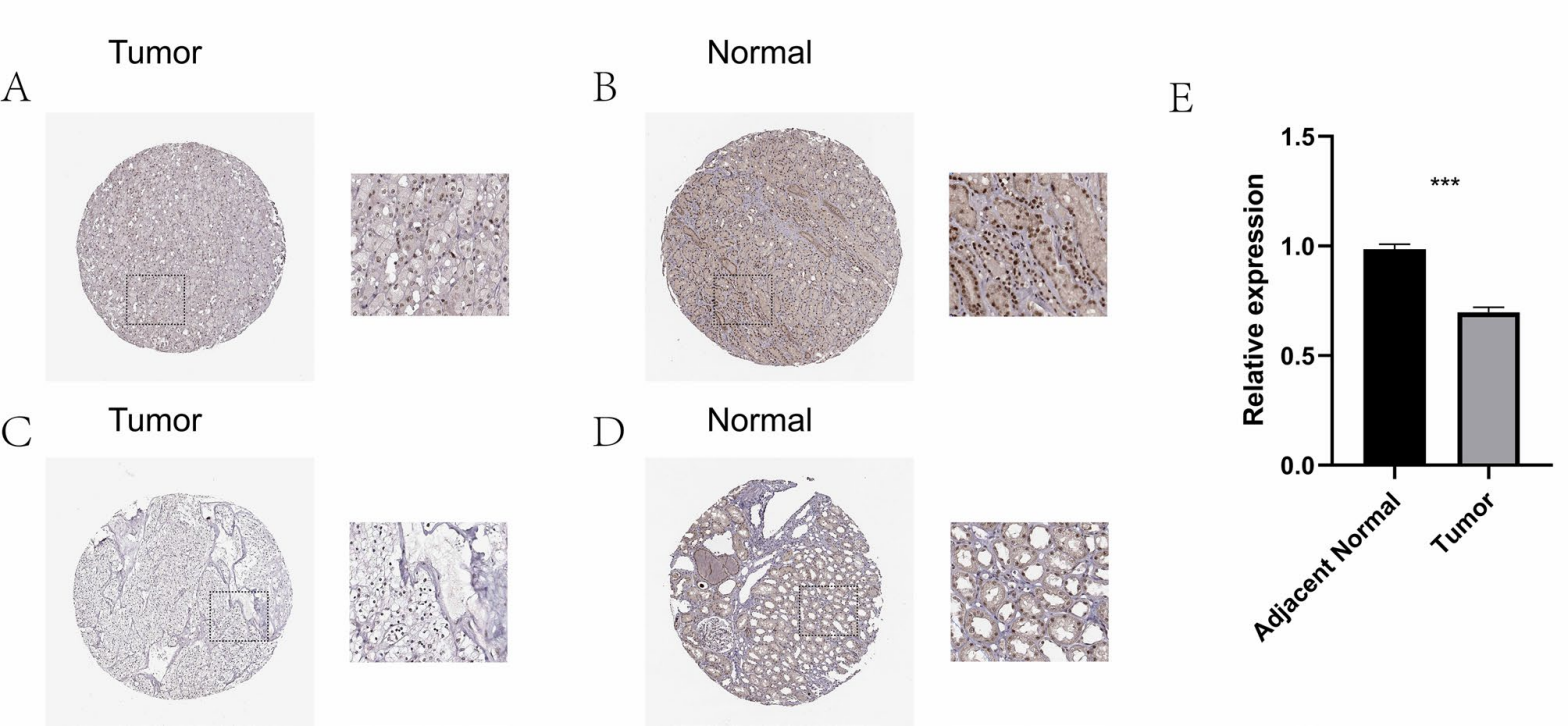
(**A**–**B**) Normal immunohistochemical results obtained from the HPA database.(**C**-**D**) Immunohistochemical results of tumor tissue obtained from the HPA database.(**E**) qRT-PCR showed the mRNA levels of PPARA in ccRCC tissue and its corresponding adjacent non-tumor tissue samples.

Immunohistochemical staining was performed to evaluate protein expression levels in tumor and adjacent normal tissues. tumor tissues exhibited relatively weak staining intensity and a lower proportion of positively stained cells (Fig. 10 A C). In contrast, adjacent normal tissues showed stronger nuclear staining and a higher density of positive cells (Fig. 10B and D). To assess the expression profile of PPARA in ccRCC, we quantified its mRNA levels in ten paired samples of primary ccRCC tissues and their corresponding adjacent non-tumorous tissues using real-time PCR. The analysis revealed a statistically significant downregulation of PPARA expression in ccRCC tissues relative to the matched adjacent non-tumor tissues (*P* < 0.05) (Fig. 10E).

## Discussion

3-MC is a representative polycyclic aromatic hydrocarbon known for its potent toxicity and classification as a classic chemical carcinogen^9^. Polycyclic aromatic hydrocarbons are ubiquitous environmental pollutants that bind to the aryl hydrocarbon receptor (AhR), playing a role in the carcinogenic activation of environmental toxins as well as in the biosynthesis and metabolism of estrogens^11,21^. The aryl hydrocarbon receptor (AhR) is a ligand-activated transcription factor critical for regulating multiple biological processes essential to human physiology. It exhibits tumor-promoting effects during carcinogenesis^22^. AhR influences key stages of tumorigenesis—initiation, promotion, progression, and metastasis—with physiologically relevant AhR ligands typically arising during disease states or heightened innate and adaptive immune responses^23^. Exposure of ccRCC cell lines to AhR ligands, such as indigo carmine or 2,3,7,8-tetrachlorodibenzo-p-dioxin (TCDD), revealed AhR pathway activation predominantly in the nuclei of high-grade ccRCC cells and tumor-infiltrating lymphocytes (TILs). Its expression levels in cancer cells and TILs correlate with pathological tumor staging and histological grading^24^. Strategic activation of AhR through selective AhR modulators can stimulate its anticancer activity by specifically targeting RB1 and AR, thereby reducing cell cycle progression and metastasis formation in ccRCC^25^. Although AhR signaling has been implicated in ccRCC, it was not directly investigated in the present study. Sbstantial evidence indicates interactions between the environmental contaminant 3-MC and ccRCC, the underlying and integrated toxic mechanisms remain unclear. SHAP interpretability analysis revealed PPARA (SHAP = 0.140) as the most influential predictor. Molecular docking further confirmed stable binding between 3-MC and the proteins encoded by these hub genes. Utilizing the TCGA database, we analyzed survival differences between high- and low-expression groups in ccRCC. Although PIK3C2G expression showed no significant impact on overall survival in ccRCC (*P* > 0.05), the other three core genes exhibited statistically significant differences (*P* < 0.05). Notably, PPARA expression demonstrated the most pronounced difference (*P* < 0.001), consistent with our previous analysis.

Our analyses of immune infiltration and functional pathways indicate that the diagnostic model is associated with coordinated changes in both the immune microenvironment and core cellular functions in ccRCC. Immune deconvolution revealed distinct differences in the infiltration of several immune cell populations, including CD8⁺ T cells, NK cells, macrophages, dendritic cells, and mast cells, between the defined risk groups. Although the strength of the correlations between the risk score and individual immune cell types was relatively modest, the consistent direction of these associations suggests that the model reflects broad immune remodeling rather than isolated alterations, which aligns with the known heterogeneity of immune infiltration in ccRCC.

Among the four genes incorporated into the model, PPARA showed more stable associations with immune cell infiltration and immune-related functional signatures, whereas the remaining genes displayed more variable correlation patterns. Consistently, ssGSEA analysis demonstrated that the overall risk score was linked to multiple immune functional states, including immune activation and regulation, implying that PPARA-associated signaling may contribute to immune modulation primarily through indirect mechanisms. This observation supports a role for PPARA in shaping immune-related processes via metabolic and functional regulation rather than through direct effects on specific immune cell subsets.

Pathway-level analyses using both GSEA and GSVA further revealed widespread disruptions in membrane transport, ionic homeostasis, and metabolic processes in tumor tissues. In particular, pathways related to ion transmembrane transport, maintenance of cellular ion balance, and oxidative phosphorylation were predominantly suppressed in ccRCC samples, indicating altered membrane integrity and metabolic reprogramming. Given the well-established role of PPARA in lipid metabolism, mitochondrial activity, and energy homeostasis, these results provide additional support for its involvement in the metabolic alterations captured by the diagnostic model^26,27^.

Single-cell transcriptomic analysis offered further resolution into the cellular context of PPARA expression. PPARA was preferentially expressed in CD24⁺ cancer stem–like cells and displayed heterogeneous distribution across multiple tumor-associated cell populations. Moreover, at the transcriptomic inference levels cell–cell communication analysis identified extensive ligand–receptor interactions involving PPARA⁺ CD24⁺ CSCs, immune cells, and stromal components, encompassing pathways related to immune regulation, extracellular matrix interactions, and angiogenesis. Together, these observations suggest that PPARA may influence tumor progression through its participation in intercellular communication networks within the tumor microenvironment.

Histologically, cytoplasmic deposits of lipids and glycogen are common in ccRCC^28^. Peroxisome proliferator-activated receptors (PPARs) are nuclear proteins that belong to the nuclear hormone receptor superfamily and serve as key mediators of lipid metabolism. They play crucial roles in biotransformation, inflammation regulation, and cancer progression^26,27^. Peroxisome proliferator-activated receptor alpha (PPAR-α) is a primary regulator of lipid metabolism. Enhancing PPAR-α expression can mitigate carcinogenic events driven by abnormal lipid metabolism in ccRCC^29^. Glypicans are members of the heparan sulfate (HS) proteoglycan family and are anchored to the cell membrane via glycosylphosphatidylinositol (GPI) anchors. To date, six family members (GPC1 to GPC6) have been identified in mammals^30^ Glypicans play roles in cellular and tissue development, morphogenesis, and cell motility. They exhibit differential expression across various cancers, functioning as tumor promoters or suppressors in a cancer-type-specific ways^31^. Related studies have also demonstrated the therapeutic potential of targeting glypican-3 (GPC3) in solid tumors, including renal carcinoma^32,33^. IK3C2G encodes PI3K-C2γ, the third distinct protein in the human Class II PI 3-kinase family, which plays a pivotal role in oncogenic transformation^34^ The PI3K/AKT/mTOR (PAM) signaling pathway is a highly conserved signal transduction network in eukaryotic cells that promotes cell survival, growth, and cell cycle progression^35^ Activation of PI3K/AKT/mTOR signaling in ccRCC significantly enhances tumorigenesis and metastasis^36^. TRPA1-associated channels play a crucial role in pain perception and chemosensation. TRPA1 expression increases sensitivity to nociception in cancer pain models^37^, which may be linked to the pain experienced in advanced (ccRCC). However, to our knowledge, no studies have specifically reported an association between TRPA1 and the occurrence or progression of ccRCC. The present study may provide preliminary evidence.

This study has several limitations. First, the quality of the original target data is limited by variations in screening criteria across databases, resulting in the absence of a unified and standardized framework. Second, the study does not control for confounding factors such as age and genetic background, which may influence the accuracy of the results. Finally, molecular docking relies on computer simulations, and further experimental verification can be conducted to supplement it. Therefore, future research should expand the study cohort to validate the identified hub genes and underlying mechanisms.

## Conclusion

In summary, this study suggests that 3-MC may be associated with ccRCC through the regulation of key genes and related signaling pathways. Molecular docking analysis further revealed the highly specific binding between 3-MC and its candidate target proteins, providing structural support for potential interactions. In vitro experiments further validated the relevant results. Our findings provide new insights into the relationship between environmental health and cancer, and the identified key genes lay an important foundation for discovering new therapeutic targets.

## Supplementary Information

Below is the link to the electronic supplementary material.

Supplementary Material 1

Supplementary Material 2

## Data Availability

All relevant data for this study are included in this manuscript. The corresponding data codes can be obtained from the corresponding author upon reasonable request.
