# Supplementary material for: Deciphering the molecular networks of 3-methylcholanthrene-induced clear cell renal cell carcinoma through multi-omics integration
Source: Sci Rep. 2026 Jan 7;16:4411. doi: 10.1038/s41598-025-34526-x (PMC12865013; doi:10.1038/s41598-025-34526-x)
Supplement: Supplementary file 1 — Supplementary Material 1 [file 41598_2025_34526_MOESM1_ESM.pdf]

Ref: 254058

Permission is granted to Scientific Reports of Springer Nature Ltd to publish both in print and digital under the CC BY 4.0 open access license the result of using KEGG and the following KEGG images in the article "Deciphering the Molecular Networks of 3-Methylcholanthrene-Induced Clear Cell Renal Cell Carcinoma through Multi-Omics Integration" written by Yuzhe Su and colleagues:

- Calcium signaling pathway (map04020)
- Bile secretion (map04976)
- Mineral absorption (map04978)
- Parathyroid hormone synthesis, secretion and action (map04928)
- Rap1 signaling pathway (map04015)
- Renin-angiotensin system (map04614)
- Endocrine and other factor-regulated calcium reabsorption (map04961)
- Neuroactive ligand signaling (map04082)
- Chemical carcinogenesis - receptor activation (map05207)
- cAMP signaling pathway (map04024)
- Gastric acid secretion (map04971)
- Taste transduction (map04742)
- Adrenergic signaling in cardiomyocytes (map04261)
- Proximal tubule bicarbonate reclamation (map04964)
- Cholinergic synapse (map04725)
- Phototransduction (map04744)
- Linoleic acid metabolism (map00591)
- Renin secretion (map04924)
- Morphine addiction (map05032)
- Central carbon metabolism in cancer (map05230)
- Purine metabolism (map00230)
- Folate transport and metabolism (map04981)
- Hormone signaling (map04081)
- PPAR signaling pathway (map03320)
- Neuroactive ligand-receptor interaction (map04080)
- Salivary secretion (map04970)
- PI3K-Akt signaling pathway (map04151)
- Ras signaling pathway (map04014)
- Retinol metabolism (map00830)
- Drug metabolism - cytochrome P450 (map00982)

subject to the condition that the original source is acknowledged by citing at least one KEGG paper.

Permission granted: Yixuan Song

Yixuan Song, Kanehisa Laboratories

Date: 11 November 2025

Copyright holder: Kanehisa Laboratories
