# Supplementary material for: Deciphering the molecular networks of 3-methylcholanthrene-induced clear cell renal cell carcinoma through multi-omics integration
Source: Sci Rep. 2026 Jan 7;16:4411. doi: 10.1038/s41598-025-34526-x (PMC12865013; doi:10.1038/s41598-025-34526-x)
Supplement: Supplementary file 2 — Supplementary Material 2 [file 41598_2025_34526_MOESM2_ESM.pdf]

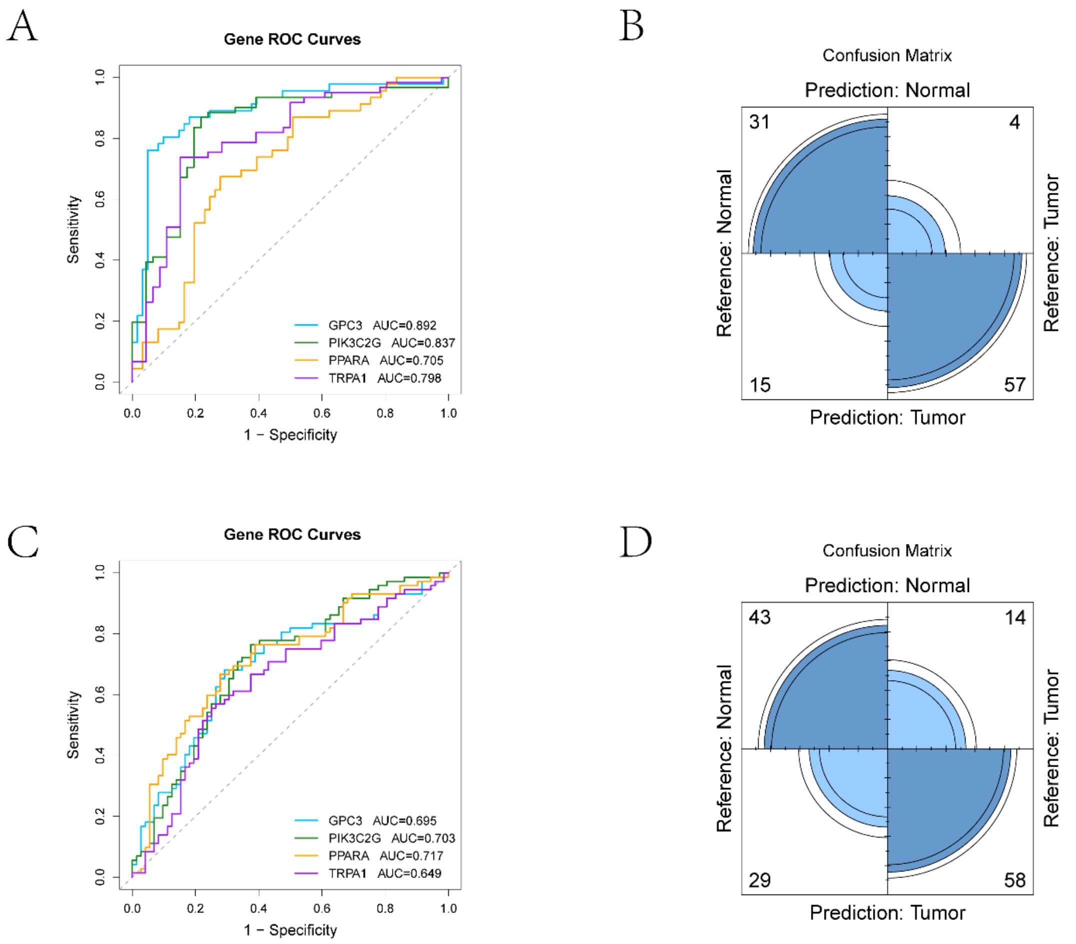

Supplementary Figure1

ROC curve and confusion matrix of the validation dataset

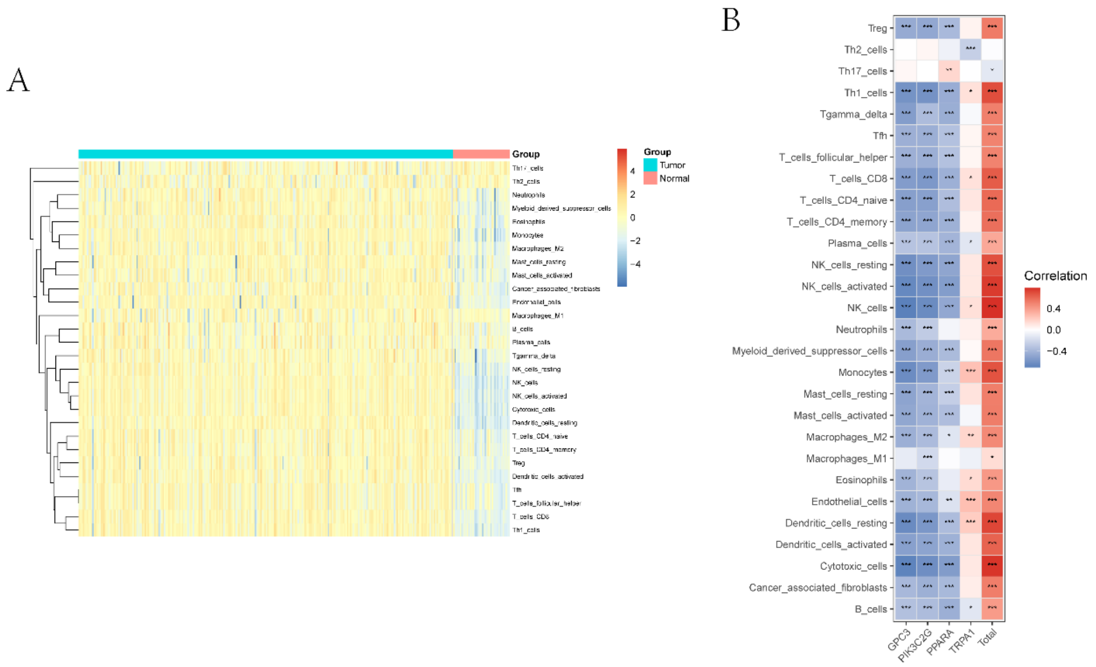

Supplementary Figure2

Assessment of immune function and comprehensive risk evaluation

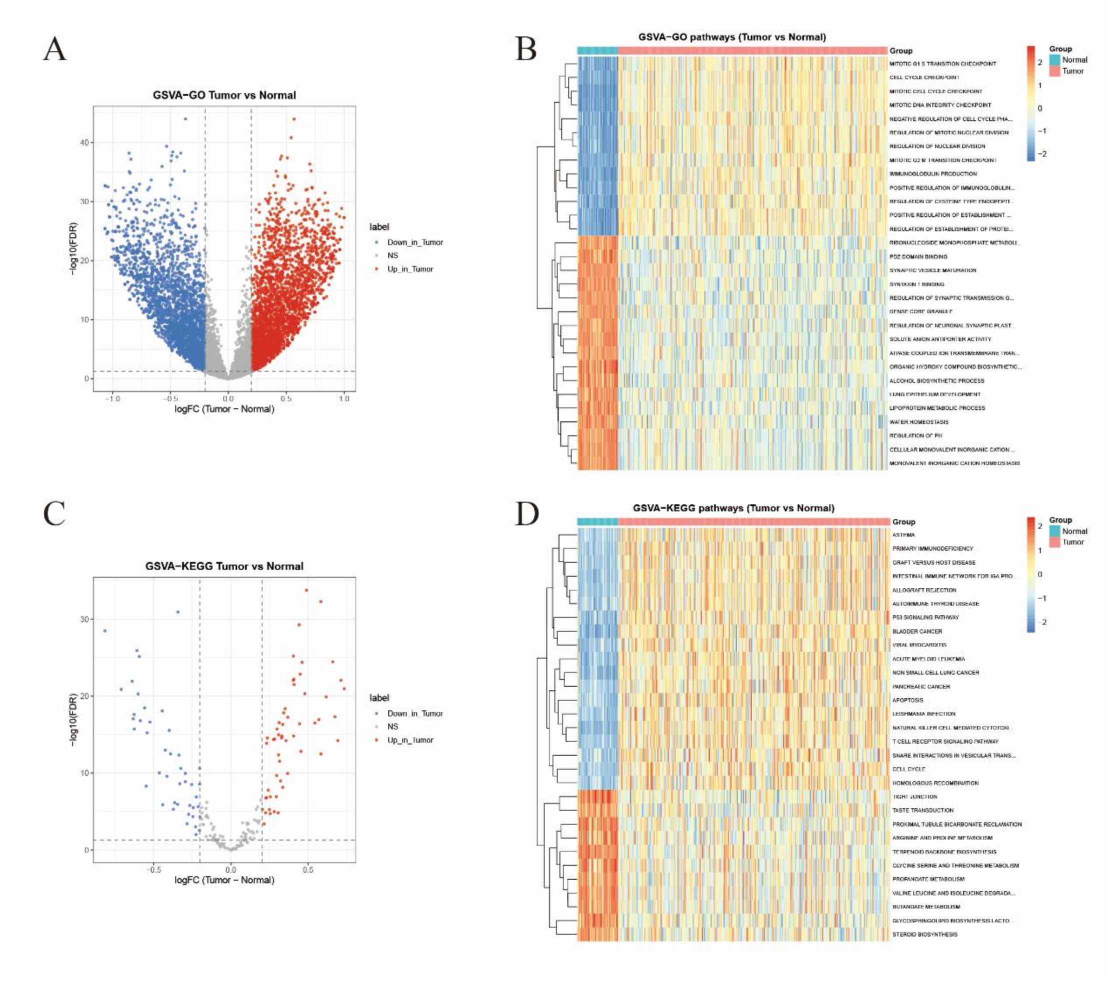

Supplementary Figure3

GSVA revealed significant activity within the examined pathways.
